# Racial and Ethnic Differences in COVID-19 Outcomes, Stressors, Fear, and Prevention Behaviors Among US Women: Web-Based Cross-sectional Study

**DOI:** 10.2196/26296

**Published:** 2021-07-12

**Authors:** Jamila K Stockman, Brittany A Wood, Katherine M Anderson

**Affiliations:** 1 Division of Infectious Diseases and Global Public Health Department of Medicine, School of Medicine University of California, San Diego La Jolla, CA United States; 2 Department of Behavioral, Social, and Health Education Sciences Rollins School of Public Health Emory University Atlanta, GA United States

**Keywords:** COVID-19, race, ethnicity, demographics, stress, fear, prevention behaviors, prevalence, cross-sectional, women, United States

## Abstract

**Background:**

In the United States, racial and ethnic minorities are disproportionately affected by COVID-19, with persistent social and structural factors contributing to these disparities. At the intersection of race/ethnicity and gender, women of color may be disadvantaged in terms of COVID-19 outcomes due to their role as essential workers, their higher prevalence of pre-existing conditions, their increased stress and anxiety from the loss of wages and caregiving, and domestic violence.

**Objective:**

The purpose of this study is to examine racial and ethnic differences in the prevalence of COVID-19 outcomes, stressors, fear, and prevention behaviors among adult women residing in the United States.

**Methods:**

Between May and June 2020, women were recruited into the Capturing Women’s Experiences in Outbreak and Pandemic Environments (COPE) Study, a web-based cross-sectional study, using advertisements on Facebook; 491 eligible women completed a self-administered internet-based cross-sectional survey. Descriptive statistics were used to examine racial and ethnic differences (White; Asian; Native Hawaiian or other Pacific Islander; Black; Hispanic, Latina, or Spanish Origin; American Indian or Alaskan Native; multiracial or some other race, ethnicity, or origin) on COVID-19 outcomes, stressors, fear, and prevention behaviors.

**Results:**

Among our sample of women, 16% (73/470) reported COVID-19 symptoms, 22% (18/82) were concerned about possible exposure from the people they knew who tested positive for COVID-19, and 51.4% (227/442) knew where to get tested; yet, only 5.8% (27/469) had been tested. Racial/ethnic differences were observed, with racial/ethnic minority women being less likely to know where to get tested. Significant differences in race/ethnicity were observed for select stressors (food insecurity, not enough money, homeschooling children, unable to have a doctor or telemedicine appointment) and prevention behaviors (handwashing with soap, self-isolation if sick, public glove use, not leaving home for any activities). Although no racial/ethnic differences emerged from the Fear of COVID-19 Scale, significant racial/ethnic differences were observed for some of the individual scale items (eg, being afraid of getting COVID-19, sleep loss, and heart racing due to worrying about COVID-19).

**Conclusions:**

The low prevalence of COVID-19 testing and knowledge of where to get tested indicate a critical need to expand testing for women in the United States, particularly among racial/ethnic minority women. Although the overall prevalence of engagement in prevention behaviors was high, targeted education and promotion of prevention activities are warranted in communities of color, particularly with consideration for stressors and adverse mental health.

## Introduction

The COVID-19 pandemic has shed light on racial and ethnic disparities in the United States [1-3]. Among 44% of US cases with known race or ethnicity, 33% were Latinx, 22% were Black, and 1.3% were American Indian or Alaskan Native (AIAN), despite accounting for 18%, 13%, and 0.7% of the US population, respectively, suggesting these groups are disproportionately affected by the COVID-19 pandemic [1]. Black individuals are almost three times more likely to be hospitalized for COVID-19 than White individuals [4]. Nationally, COVID-19 mortality is 80% higher for Black individuals and over 50% higher for Latinx individuals, relative to White individuals [5].

Contributing factors to racial and ethnic disparities in COVID-19 outcomes include social and structural vulnerabilities and pre-existing health conditions, affecting socially marginalized populations [6,7]. Residing in high-poverty areas, being underinsured or uninsured, and having limited access to health care likely contribute to limited COVID-19 testing, resulting in late presentation with more advanced disease [8-10]. Historically, Black individuals and AIAN individuals have endured systemic racism and discrimination, a source of structural inequities [10], that has created deep mistrust of medical institutions and unequal access to care and treatment [11]. Ethnic minority groups are overrepresented in essential service industries, limiting the ability to shelter at home and increasing the likelihood of low wages, unpaid sick leave, or limited workplace protections [10,12]. The physical environment also may increase COVID-19 risk through residential segregation, residing in densely populated areas, and multigenerational households; the resulting physical crowding contributes to increased viral transmission [7,13]. Ethnic minority groups are disproportionately affected by chronic medical conditions, which may worsen COVID-19 outcomes [2]. Moreover, toxic stress resulting from racial and social inequities has been magnified during the pandemic, with implications for poor biological function and adverse physical and mental health outcomes [11,14]. This culminates in Black, Latinx, and AIAN groups shouldering greater disease burden and the unfolding spread of COVID-19 in areas with larger populations of ethnic minorities [8].

The continuation of the COVID-19 pandemic has led the US government to recommend prevention behaviors, including staying at home except for essential activities, physical distancing from nonhousehold members, wearing a face mask in public, avoiding nonhousehold groups, and frequent handwashing [15]. Compliance with these recommendations has been variable [16-19]. A May 2020 Centers for Disease Control and Prevention (CDC) survey found that 91.5% of participants had been to a public area in the previous week, 77.3% stayed home except for essential activities, and 75.1% and 10.8% were “always” and “often” avoiding groups of 10 or more people, respectively [17]. Further, physical distancing was maintained “always” by 58.2% and “often” by 21.3% of respondents, and 60.3% “always” and 13.8% “often” wore a face mask in public [17]. A survey conducted in early April 2020 found that 87% of participants were physically distancing in public, while only 50% used face masks in public; older age, female gender, and financial security were associated with adherence, with no differences by race or ethnicity, possibly due to overrepresentation of White participants [16]. Other assessments of racial and ethnic differences in prevention vary. In one study, Black adults knew less about symptoms and routes of transmission of COVID-19, left their homes more frequently, and handwashed less often than White adults [19]; in another, Black adults were less likely to maintain physical distance or avoid groups compared to their White counterparts but more likely to stay home except for essential activities, more likely to “always” or “often” wear a face mask in public, and equally likely to have been in a public area in the preceding week [17].

Given the lack of published findings on COVID-19 outcomes by race/ethnicity among women in the United States, our paper describes the prevalence and racial/ethnic differences among COVID-19 outcomes, stressors, fear, and prevention behaviors among an ethnically diverse sample of US adult women.

## Methods

### Study Population

Between May and June 2020, we conducted the Capturing Women’s Experiences in Outbreak and Pandemic Environments (COPE) Study, a web-based cross-sectional study of COVID-19 outcomes, testing, and prevention behaviors among adult women in the United States. Our study included participants who were 18 years or older, cisgender or transgender female, living in the United States, and able to understand English. We excluded participants who did not reside within the United States, were younger than 18 years, were male, or did not understand English.

### Recruitment and Enrollment

Recruitment occurred through Facebook advertisements, designed to recruit adult women currently residing in the United States. We aimed to collect at least 400 survey responses. Interested women accessed a digital consent form within the REDCap (Research Electronic Data Capture) survey platform. The consent form included the purpose of the study and participant risk, burden, rights, and compensation. Participants who provided documented consent were directed to a screening survey. Screening questions included age, gender, and state of residence. Of 682 users who clicked on an advertisement, 633 (92.8%) provided informed consent and answered screener questions. Among the 633 participants, 7 participants were not eligible for the study. Eligible participants (n=626) were directed to the survey, which took 15 to 20 minutes to complete. Of 626 eligible participants, 491 (78% response rate) completed the full web-based survey. Participants received a US $20 Amazon e-gift card via email. The COPE Study procedures were approved by the University of California, San Diego Human Research Protections Program Institutional Review Board.

### Survey Measures

#### Sociodemographic Characteristics

Race/ethnicity were self-reported by using the following categories: White (eg, German, Irish, English, Italian, Polish, or French); Asian, Native Hawaiian, or other Pacific Islander (API); Black (eg, African American or Black Caribbean); Hispanic, Latina, or Spanish origin (eg, Mexican or Mexican American, Puerto Rican, Cuban, or Dominican); AIAN (eg, Navajo nation, Blackfeet tribe, or Mayan); and multiracial or some other race, ethnicity, or origin. “Other race, ethnicity, or origin” included 7 women who self-identified as Middle Eastern or North African. Other demographics included age, education, employment status, sexual orientation, relationship status, parenthood, household composition and type of occupants, type of residential community (eg, urban or rural), current living situation, and household income.

#### COVID-19–Related Outcomes

We modified COVID-19 measures developed by the World Health Organization (WHO) for rapid behavioral studies about COVID-19 [20]. We assessed whether participants and close others (eg, family, friend, partner, or coworker) had COVID-19 symptoms, were tested, knew where to get tested, were diagnosed, were hospitalized, or died from COVID-19.

#### COVID-19 Stressors and Fear

We assessed nine COVID-19 stressors as outlined by the WHO [20]. Examples included food insecurity, insufficient rent, and caregiver status. We employed the Fear of COVID-19 Scale, a 7-item self-reported measure of an individual’s fear of COVID-19 [21]. Items include fear of dying of COVID-19 and loss of sleep due to worrying about COVID-19. Responses followed a 5-point Likert scale from “strongly disagree” to “strongly agree.” Individual items were assessed, and the overall score was calculated by summing all 7 items (range 7-35; Cronbach alpha .90).

#### COVID-19 Prevention Behaviors

COVID-19 prevention behaviors examined were: washing hands, avoiding touching face, using disinfectants (including hand sanitizer), staying home except for essential activities, covering mouth when coughing, physical distancing from nonhousehold members, physical distancing if sick, isolating oneself if sick, public face mask use, public glove use, avoiding crowds, and not leaving home [20]. Responses to each were dichotomized.

### Data Analysis

Excluding participants without reported race/ethnicity, the sample was 473 women. We computed descriptive statistics of all variables overall and by race/ethnicity. We report medians and IQRs for nonnormally distributed continuous variables and frequencies and proportions for categorical variables. For race/ethnicity comparisons, we used Kruskal-Wallis tests and chi-square tests. If subgroups had expected values less than 5, Fisher exact test or a Monte Carlo estimate for Fisher exact *P* value was used. Individual pairwise comparisons by race/ethnicity were conducted when the Kruskal-Wallis test or chi-square test had a *P*<.05. The Dwass-Steel-Critchlow-Fligner test was used when the Kruskal-Wallis test was significant (*P*<.05), and individual chi-square tests between each of the racial/ethnic groups were examined when the overall chi-square was significant (*P*<.05). To account for potential confounding, COVID-19–related outcomes, stressors, and prevention behaviors that were significantly associated in bivariate analyses were included in multivariable binary logistic regressions. Each regression was adjusted for age, education, income, and type of residential community (urban, suburban, rural), with race as the independent variable and the outcome, stressor, or prevention behavior as the dependent variables. All analyses were performed using SAS, version 9.4 (SAS Institute).

## Results

### Sociodemographics

Of 473 women, 51% (n=241) were White, 13.5% (n=64) API, 12.7% (n=60) Black or African American, 10.1% (n=48) Hispanic or Latinx, 5.7% (n=27) AIAN, and 7% (n=33) multiracial or “other” (Multimedia Appendix 1). The median age was 33 years, with White women significantly older than API, AIAN, and multiracial or other women. Most women had completed some college or graduate school (377/463, 79.7%). Approximately 40% (175/473) of women were unemployed. Approximately 70% (323/471) were in a relationship, and 45% (208/471) had children.

Approximately 46% (211/461) of women lived alone. Of 250 living with others, 55.2% (n=138) and 53.2% (n=133) lived with their partner and family members, respectively. The median number of children in the household was 2. Almost 44% (193/447) had a household income of US $50,000 or more, with White individuals more likely than all racial/ethnic groups, except Latinx. Almost 50% (212/466) of women lived in an urban environment and 17.6% (82/466) in a rural environment.

### COVID-19 Outcomes

Approximately 16% (73/470) of women reported COVID-19 symptoms; the highest prevalence was among White (45/240, 18.8%) and multiracial or other (7/33, 21.2%) women, and the lowest prevalence was among API women (4/63, 6.4%; Multimedia Appendix 2). Although 51.4% (227/442) of women knew where to get tested for COVID-19, only 5.8% (27/469) had been tested. White women were more likely to know where to get tested than API, Latinx, and AIAN women (*P*<.001, *P*=.01, and *P*=.01, respectively), and Black women more likely than API and AIAN women (*P*=.01 and *P*=.03, respectively). A total of 2% (8/469) of women were diagnosed with COVID-19, of which 50% (4/8) were hospitalized; 22% (18/82) of women were concerned that they were exposed to COVID-19 by the people they know. No significant differences by race/ethnicity were found for either.

A total of 18% (82/469) of women knew someone diagnosed with COVID-19, with White and Black women more likely than API women (*P*=.02 for both); 67.1% (55/82) identified a diagnosed friend, and 42.7% (35/82) identified diagnosed family. Of the 82 women, 42.7% (n=35) knew someone hospitalized, with Black women more likely than White women (*P*<.001); family members (16/35, 45.7%) and friends (14/35, 40.0%) were the most frequently reported relationships. Black women were more likely than White women to report a family member hospitalized (*P*=.01). Of the 82 women, 22% (n=18) knew someone who had died of COVID-19; most were a friend (8/18, 44.4%) or family member (6/18, 33.3%).

### COVID-19 Stressors and Fear

Racial/ethnic differences emerged for four COVID-19 stressors (Multimedia Appendix 3). A total of 17% (82/473) of women experienced food insecurity. White women were less likely than API, Black, Latinx, AIAN, and multiracial or other race women to not have enough food (*P*<.001, *P*<.001, *P*=.03, *P*<.001, and *P*=.002, respectively); API women were more likely than Latinx women (*P*=.046) to not have enough food. Approximately 20% (91/473) did not have enough money for rent; White women were less likely to not have enough money for rent than API, Black, Latinx, and AIAN women (*P*<.001, *P*<.001, *P*=.03, and *P*<.001, respectively). Of 20.9% (99/473) who reported homeschooling children, White women were more likely than API and AIAN women (*P*=.03 and *P*=.003, respectively), and Black and Latinx women were more likely than AIAN women (*P*=.004 and *P*=.01, respectively). Of 28.1% (133/473) unable to have a doctor or telemedicine appointment, White women were more likely than API and AIAN women (*P*=.003 and *P*=.03, respectively). For the remaining stressors, 36.6% (173/473) of women reported loss of income, 15.4% (73/473) were essential employees, 4.7% (22/473) were unable to obtain medication, and 9.9% (47/473) were caregivers for sick family without or with (15/473, 3.2%) COVID-19.

Although no racial/ethnic differences emerged for the Fear of COVID-19 Scale, significant differences were observed for individual items. Higher agreement with the statement “I am very afraid of coronavirus” was found among White women compared to API and multiracial or other women (*P*=.01 and *P*=.02, respectively). White women reported more agreement with becoming nervous or anxious when watching media about COVID-19 compared to API women (*P*=.001). Latinx women agreed with experiencing clammy hands when thinking about COVID-19 more than White and multiracial or other women (*P*=.02 for both); AIAN women agreed more than White, Black, and multiracial or other women (*P*=.002, *P*=.03, and *P*=.02, respectively). AIAN women agreed more with sleep loss due to worrying about getting COVID-19 compared to White, Black, and multiracial or other women (*P*=.01, *P*=.002, and *P*=.01, respectively); API women agreed more than Black women (*P*=.02). AIAN women also had more agreement with experiencing their heart racing when thinking about getting COVID-19 compared to Black women (*P*=.04).

### COVID-19 Prevention Behaviors

Significant differences in race/ethnicity were observed for four COVID-19 prevention behaviors (Multimedia Appendix 4). Of 95.6% (452/473) of women reporting handwashing with soap, White and API women were more likely than AIAN women (*P*=.002 and *P*=.02, respectively). Of 60.0% (284/473) who self-isolated if sick, API women were more likely than White, Black, and multiracial or other women (*P*=.01, *P*=.004, and *P*=.002, respectively). Of 40.2% (190/473) who publicly used gloves, API, Black, and AIAN women were more likely than White women (*P*<.001, *P*=.047, and *P*<.001, respectively); API women were more likely to publicly use gloves than Latinx and multiracial or other women (*P*<.001 for both); AIAN women were more likely to publicly use gloves than Black, Latinx, and multiracial or other women (*P*=.02, *P*=.01, and *P*<.001, respectively). Of 43.6% (206/473) who did not leave home at all, White women were less likely than API, AIAN, and multiracial or other women (*P*<.001, *P*=.001, and *P*=.04, respectively); API women were more likely to not leave home at all than Black and Latinx women (*P*=.01 and *P*=.001, respectively); AIAN women were more likely to not leave home at all than Latinx women (*P*=.02). Approximately 80% (374/473) of women avoided face touching, 87.1% (412/473) used disinfectants, 88.6% (419/473) stayed home except for essential activities, 82.2% (389/473) covered mouths when coughing, 86.5% (409/473) physically distanced from nonhousehold members, 59.8% (283/473) physically distanced from others if sick, 79.7% (377/473) publicly used a face mask, and 81.8% (387/473) avoided crowds.

To account for potential confounding, COVID-19–related outcomes, stressors, and prevention behaviors that were significantly associated with race or ethnicity in bivariate analyses were identified for inclusion in multivariable binary logistic regressions. Each regression was adjusted for age, education, income, and type of residential community (urban, suburban, rural). Results of these regressions are available in Multimedia Appendix 5 and confirm that there are statistically significant differences in all COVID-19–related outcomes, stressors, and prevention behaviors by race and ethnicity in comparison to the reference group, after accounting for potential confounders.

## Discussion

### Principal Findings

To our knowledge, this is the first study to describe COVID-19 outcomes, stressors, fear, and prevention behaviors among a sample of US women and to examine racial/ethnic differences. Our data showed that 1 in 6 women had COVID-19 symptoms. Troublingly, although 51.4% (227/442) of women knew where to get tested for COVID-19 and 22.0% (18/82) were concerned about possible exposure to COVID-19, only 5.8% (27/469) had been tested.

White women were the most likely to know where to be tested for COVID-19, despite experiencing a lower burden of COVID-19 among family and friends compared to Black women. This may be due to persistent medical mistrust due to legacies of abuse, mistreatment [22,23], and discrimination as an antecedent to medical mistrust [24]. Social and economic inequalities shape and sustain medical mistrust, particularly among populations who experience staggering health disparities [23]. Further, this may be reflective of an inability to access testing due to structural inequities [10], compounded by employment as essential workers [10,12], limiting available sick leave for testing and support for accessing health care. Given this, it is imperative to engage underserved populations in education and testing through community mobilization, social network, and peer-led engagement approaches.

A total of 18% (82/469) of women knew someone close to them diagnosed with COVID-19—often family or friends—with White and Black women being more likely than API women. Black women were more likely than White women to know someone hospitalized for COVID-19 and report them as family. This demonstrates a high burden of COVID-19 morbidity and mortality among Black families and their social circles; further, this implies a potential burden of adverse mental health associated with loss and trauma.

Racial/ethnic differences were observed for select stressors. White women were less likely than all others to lack food and money for rent, possibly because White women were more likely to have a household income over US $50,000, ensuring the ability to sustain food and housing expenses. White women were also more likely than API and AIAN women to be unable to go to the doctor or have a telemedicine appointment and report homeschooling children. Collectively, plausible explanations are that White women had competing responsibilities due to living with other family members and having a higher median number of household residents, which prevented in-person appointments with their doctor. Additionally, White women may have had privacy concerns with engaging in a phone or video telemedicine visit with their provider due to more family members or household residents being present.

Although significant differences by race/ethnicity were not identified by summed scores for the Fear of COVID-19 Scale, there were significant differences in many of the individual items, showing dimensionality within experiences of fear of COVID-19. White women consistently agreed with statements regarding psychological experiences of fear (being “very afraid” or “nervous or anxious”) relating to COVID-19, while API, Latinx, and AIAN women consistently endorsed agreement with physical manifestations of fear (“hands become clammy,” “cannot sleep,” “heart races”). Black women did not fall into these patterns, endorsing most agreement with being very afraid and experiencing anxiety relating to media about COVID-19. This may reflect differential conceptualization of fear and stress, including antidisclosure socialization and stigma around mental health [25-27] or increased symptomatology associated with adverse mental health resulting from cumulative or toxic stress [11,14].

Overall, we found high prevalence of implementation of CDC-recommended prevention behaviors. Prevalence of staying home except for essential activities was 88.6% (419/473) compared to a rate of 77.3% among US adults surveyed in May 2020 [17]. Likewise, 79.7% (377/473) of women in our study reported using a face mask, slightly higher than 74.1% of US adults surveyed in May [17]. Previous findings demonstrated decreased prevalence in physical distancing behavior from 87% in April to 79.5% in May [16,17]; in our study, 86.5% (409/473) of women reported this behavior. These differences may reflect increased uptake of prevention behaviors among women, compared to a sample of both men and women; this is consistent with findings that female gender is associated with adherence to prevention behaviors [16].

Self-isolation at home if sick was more likely among API women, possibly due to customary traditions of preventing illness. Handwashing with soap was more likely among White and API women than AIAN women. This may be due to White and API women’s increased access to education on preventing COVID-19, primarily disseminated through television and the internet. Of further importance is the possibility of performative practice of prevention behaviors among API women, in response to COVID-19–related xenophobia; individuals of Asian descent have been the targets of hate speech and crime based on the origination of COVID-19 in China [28]. Among AIAN populations, access to the internet is limited [29], and this group is further disadvantaged due to low rates of health literacy [30], limiting knowledge of prevention behaviors. Native patient navigators, similar to community health workers [31], are an avenue for education on COVID-19 prevention in AIAN communities.

API women were more likely than Black and Latinx women to not leave home for any activities, possibly due to API increased partner cohabitation, allowing them to remain indoors and rely on a partner for essential activities. Consistent with existing data, Black and Latinx populations are overrepresented in essential service industries, limiting the ability to shelter at home [10,12]. Because White women had more household residents compared to AIAN women and less partner cohabitation compared to API women, it is plausible that White women were less likely to stay home, due to the need for essential items such as food and medicine.

Supplemental analyses to assess confounding confirmed statistically significant variation between racial/ethnic groups in binary logistic regressions after adjustment for age, education, income, and type of residential community, in comparison to White participants. All adjusted odds ratios were in the expected directions and consistent with bivariate findings. However, the variation in relationship between race/ethnicity and COVID-19–related outcomes, stressors, and prevention behaviors indicate a need for further research to assess the nuanced experiences of women of all racial and ethnic identities, which was beyond the scope of this analysis, to ensure culturally responsive and appropriate public health programming.

### Limitations and Strengths

The primary limitation of our research was the use of nonprobability sampling methods with recruitment through online social media, resulting in a lack of generalizability of our study findings. Thus, our sample is not representative of adult women in all US states. Our sample was younger to middle-aged, primarily resided in urban environments, and did not consist of racial/ethnic groups and other demographics such as age, education, and income that were proportionate to each US state population’s demographics. Specifically, 17.6% (82/466) of our sample resided in a rural environment, slightly underrepresenting the 19.3% of the US population in rural areas [32]. Although ethnically diverse, our sample underrepresents Latinx women while approximating the US population of Black women. Yet, API and AIAN women are overrepresented in our sample, which may add to the utility of these findings, given the underrepresentation of these groups in research. Finally, only women with access to the internet were able to complete the survey. However, 75% of US adult women use Facebook, with three-quarters accessing Facebook daily [33], indicating that the sampling frame encompassed a significant portion of the US population.

Limitations notwithstanding, our data highlight racial/ethnic differences in COVID-19–related outcomes among adult US women. Our ability to recruit an ethnically diverse sample of women that comprised 41 US states and Washington, DC using online recruitment methods within a short time frame demonstrates the willingness of women to share their experiences on COVID-19. Future studies are needed that use probability sampling methods and longitudinal cohort study designs to examine trends over time in racial/ethnic disparities of COVID-19 outcomes and elucidate underlying factors fueling these disparities among a representative sample of adult US women.

### Conclusions

The lower knowledge of testing availability, actualized testing, and prevention behaviors among Black, Latinx, and AIAN women compared to their White and API counterparts will serve to exacerbate the high rates of COVID-19 mortality already demonstrated among these populations. Meanwhile, stressors, physical manifestations of fear, and adverse mental health associated with loss of family and friends to COVID-19 may compound existing toxic stress, culminating in increasingly negative chronic health outcomes. It is vital that public health interventions for COVID-19 focus on these communities, ensuring testing, health care, and support services—including mental health, economic, and social services—are accessible and acceptable, rather than allowing for disparities to persist and worsen.

## Appendix

Multimedia Appendix 1 Demographic characteristics by racial/ethnic group among adult women in the United States (N=473).

Multimedia Appendix 2 COVID-19 outcomes by racial/ethnic group among adult women in the United States (N=473).

Multimedia Appendix 3 COVID-19 stressors and fear by racial/ethnic group among adult women in the United States (N=473).

Multimedia Appendix 4 COVID-19 public health prevention behaviors by racial/ethnic group among adult women in the United States (N=473).

Multimedia Appendix 5 Unadjusted and adjusted binary logistic regression models of COVID-19–related outcomes, stressors, and prevention behaviors and racial or ethnic group among adult women in the United States (N=473).

## Abbreviations

AIAN: American Indian or Alaskan Native
API: Asian, Native Hawaiian, or other Pacific Islander
CDC: Centers for Disease Control and Prevention
COPE: Capturing Women’s Experiences in Outbreak and Pandemic Environments
REDCap: Research Electronic Data Capture
WHO: World Health Organization
