# Supplementary material for: Racial and Ethnic Differences in COVID-19 Outcomes, Stressors, Fear, and Prevention Behaviors Among US Women: Web-Based Cross-sectional Study
Source: J Med Internet Res. 2021 Jul 12;23(7):e26296. doi: 10.2196/26296 (PMC8276781; doi:10.2196/26296)
Supplement: Multimedia Appendix 1 [file jmir_v23i7e26296_app1.pdf]

Multimedia Appendix 1. Demographic characteristics by racial/ethnic group among adult women in the United States (N=473).[a]

Characteristic	Overall (N=473)	White (n=241)	API[b] (n=64)	Black (n=60)	Latinx (n=48)	AIAN[c] (n=27)	Multiracial or other (n=33)	*P* value
Age (years), median (IQR)	33 (28-40)	36 (30-46)[d,e,f]	31 (28.5-33)[d]	32 (27-38.5)	32.5 (28.5-38)	29 (26-33)[e]	29 (23-37)[f]	<.001
**Education, n (%)**								.01
Completed high school/GED[g] or less	86 (18.6)	32 (13.3)	17 (27.9)	11 (19.3)	15 (33.3)	4 (15.4)	7 (21.2)	
Some or completed trade school, vocational school, or college	256 (55.3)	130 (53.9)	36 (59.0)	31 (54.4)	22 (48.9)	18 (69.2)	19 (57.6)	
Some or completed graduate school	121 (26.1)	79 (32.8)[h,i]	8 (13.1)[h]	15 (26.3)	8 (17.8)[i]	4 (15.4)	7 (21.2)	
Unemployed, n (%)	175 (37.0)	84 (34.9)[j]	34 (53.1)[j,k,l]	22 (36.7)	14 (29.2)[k]	6 (22.2)[l]	15 (45.5)	.03
Unemployed as of March 1, 2020[m], n (%)	65 (37.1)	26 (31.0)	16 (47.1)	9 (40.9)	3 (21.4)	2 (33.3)	9 (60.0)	.17
**Sexual orientation, n (%)**								.20
Heterosexual	383 (82.0)	190 (79.2)	56 (91.8)	48 (81.4)	40 (85.1)	25 (92.6)	24 (72.7)	
Gay or lesbian	20 (4.3)	13 (5.4)	1 (1.6)	3 (5.1)	1 (2.1)	2 (7.4)	0 (0)	
Bisexual	48 (10.3)	26 (10.8)	4 (6.6)	7 (11.9)	5 (10.6)	0 (0)	6 (18.2)	
Other[n]	16 (3.4)	11 (4.6)	0 (0)	1 (1.7)	1 (2.1)	0 (0)	3 (9.1)	
In a current relationship, n (%)	323 (68.6)	163 (67.9)[o,p]	48 (75.0)[q,r]	30 (50.0)[o,q,s,t]	34 (70.8)[s,u]	26 (100.0)[p,r,t,u,v]	22 (66.7)[v]	<.001

Characteristic		Overall (N=473)	White (n=241)	API[b] (n=64)	Black (n=60)	Latinx (n=48)	AIAN[c] (n=27)	Multiracial or other (n=33)	*P* value
**Relationship status[w], n (%)**									<.001
	Casual dating	43 (13.5)	5 (3.1)[d,p,x,y]	11 (23.4)[d,z,aa]	8 (27.6)[x,ab]	2 (6.1)[z,ab,ac,ad]	14 (53.9)[p,aa,ac,ae]	3 (14.3)[y,ad,ae]	
	Committed, not married	99 (31.1)	50 (30.9)	15 (31.9)	9 (31.0)	6 (18.2)	7 (26.9)	12 (57.1)	
	Married	170 (53.5)	102 (63.0)	21 (44.7)	11 (37.9)	25 (75.8)	5 (19.2)	6 (28.6)	
	Separated[af]	6 (1.9)	5 (3.1)	0 (0)	1 (3.5)	0 (0)	0 (0)	0 (0)	
Has children, n (%)		208 (44.2)	128 (53.1)[f,h,p]	19 (30.2)[h]	26 (44.1)[ag]	23 (48.0)[u]	3 (11.1)[p,u,ag]	9 (27.3)[f]	<.001
**Household composition, median (IQR)**									
	Number of children <18 years in household	2 (1-3)	2 (1-3)	3 (2-4)	2 (1-3)	1 (1-3)	3 (1-3)	2 (2-4)	.32
	Number of children ≥18 years in household	2 (0-3)	1.5 (0-3)	5 (5-5)	2 (1-3)	1 (0-3)	—[ah]	3 (0-3)	.50
	Number of people living with	3 (2-4)	3 (2-4)[ai]	3 (2-4)	4 (3-4)[aj]	3 (2-5)	2 (2-2.5)[ai,aj]	2 (2-3)	.002
**Household composition: type of occupants[ak], n (%)**									
	Only self	211 (45.8)	110 (45.6)	20 (33.9)	33 (56.9)	25 (54.4)	10 (41.7)	13 (39.4)	.14
	Significant other, partner, or spouse	138 (55.2)	68 (51.9)[j]	30 (76.9)[j,al,am]	9 (36.0)[al,an]	14 (66.7)[an]	8 (57.1)	9 (45.0)[am]	.02
	Other family members	133 (53.2)	85 (64.9)[d,e,ao]	12 (30.8)[d,ap]	16 (64.0)[ap,aq,ar]	10 (47.6)	4 (28.6)[e,aq]	6 (30.0)[ao,ar]	<.001

Characteristic		Overall (N=473)	White (n=241)	API[b] (n=64)	Black (n=60)	Latinx (n=48)	AIAN[c] (n=27)	Multiracial or other (n=33)	*P* value
	Friends	17 (6.8)	7 (5.3)	2 (5.1)	2 (8.0)	2 (9.5)	2 (14.3)	2 (10.0)	.56
	Roommates	22 (8.8)	10 (7.6)[f]	3 (7.7)	1 (4.0)[as]	0 (0)[at]	2 (14.3)	6 (30.0)[f,as,at]	.03
**Type of residential community, n (%)**									<.001
	Urban or city	212 (45.5)	77 (32.1)[d,f,x,au]	37 (59.7)[d]	38 (65.5)[x]	24 (50.0)	17 (65.4)[au]	19 (59.4)[f]	
	Rural	82 (17.6)	54 (22.5)	5 (8.1)	7 (12.1)	9 (18.8)	3 (11.5)	4 (12.5)	
	Suburban	172 (36.9)	109 (45.4)	20 (32.3)	13 (22.4)	15 (31.3)	6 (23.1)	9 (28.1)	
**Current living situation, n (%)**									.28
	Living in own place, room, apartment, or house	412 (91.0)	214 (91.5)	54 (91.5)	49 (86.0)	40 (90.9)	24 (88.9)	31 (96.9)	
	Temporarily doubled up with others in someone else's house	31 (6.8)	16 (6.8)	5 (8.5)	4 (7.0)	4 (9.1)	2 (7.4)	0 (0)	
	Other living situation[av]	10 (2.2)	4 (1.7)	0 (0)	4 (7.0)	0 (0)	1 (3.7)	1 (3.1)	
**Household income (US $), n (%)**									<.001
	<30,000	139 (31.1)	45 (20.0)	34 (54.0)	20 (35.7)	12 (26.7)	14 (51.9)	14 (45.2)	
	30,000-49,999	115 (25.7)	51 (22.7)	15 (23.8)	17 (30.4)	14 (31.1)	10 (37.0)	8 (25.8)	
	≥50,000	193 (43.2)	129 (57.3)[d,o,p,ao]	14 (22.2)[d,k]	19 (33.9)[o]	19 (42.2)[k,aw]	3 (11.1)[p,aw]	9 (29.0)[ao]	

[a]Certain percentages may reflect denominators smaller than the n value given in the column heading. These discrepancies are due to missing data.

[b]API: Asian, Native Hawaiian, or other Pacific Islander.

[c]AIAN: American Indian or Alaskan Native.

[d]The difference between White and API women is statistically significant at $P<.001$.

[e]The difference between White and AIAN women is statistically significant at $P=.01$.

[f]The difference between White and multiracial/other race women is statistically significant at $P=.01$.

[g]GED: General Educational Development.

[h]The difference between White and API women is statistically significant at $P=.001$.

[i]The difference between White and Latinx women is statistically significant at $P=.002$.

[j]The difference between White and API women is statistically significant at $P=.01$.

[k]The difference between API and Latinx women is statistically significant at $P=.01$.

[l]The difference between API and AIAN women is statistically significant at $P=.01$.

[m]Percentages correspond with the 175 women who said they were unemployed.

[n]Includes 7 unsure and 9 other.

[o]The difference between White and Black women is statistically significant at $P=.01$.

[p]The difference between White and AIAN women is statistically significant at $P<.001$.

[q]The difference between API and Black women is statistically significant at $P=.004$.

[r]The difference between API and AIAN women is statistically significant at $P=.004$.

[s]The difference between Black and Latinx women is statistically significant at $P=.03$.

[t]The difference between Black and AIAN women is statistically significant at $P<.001$.

[u]The difference between Latinx and AIAN women is statistically significant at $P=.001$.

[v]The difference between AIAN and multiracial/other race women is statistically significant at $P=.001$.

[w]Percentages correspond with the 318 women who said they were in a current relationship and answered this question.

[x]The difference between White and Black women is statistically significant at $P<.001$.

[y]The difference between White and multiracial/other race women is statistically significant at $P=.004$.

[z]The difference between API and Latinx women is statistically significant at $P=.02$.

[aa]The difference between API and AIAN women is statistically significant at $P=.02$.

[ab]The difference between Black and Latinx women is statistically significant at $P=.01$.

[ac]The difference between Latinx and AIAN women is statistically significant at $P<.001$.

[ad]The difference between Latinx and multiracial/other race women is statistically significant at $P=.002$.

[ae]The difference between AIAN and multiracial/other race women is statistically significant at $P=.02$.

[af]Includes separated, not seeing someone else, and seeing someone casually.

[ag]The difference between Black and AIAN women is statistically significant at $P=.003$.

[ah]AIAN women reported 0 children 18 years or older currently staying in their household.

[ai]The difference between White and AIAN women is statistically significant at $P=.02$.

[aj]The difference between Black and AIAN women is statistically significant at $P=.02$.

[ak]Percentages for significant others, other family members, friends, and roommates correspond with the 250 women who reported currently living or staying with other people.

[al]The difference between API and Black women is statistically significant at *P*=.001.

[am]The difference between API and multiracial/other race women is statistically significant at *P*=.01.

[an]The difference between Black and Latinx women is statistically significant at *P*=.04.

[ao]The difference between White and multiracial/other race women is statistically significant at *P*=.003.

[ap]The difference between API and Black women is statistically significant at *P*=.01.

[aq]The difference between Black and AIAN women is statistically significant at *P*=.03.

[ar]The difference between Black and multiracial/other race women is statistically significant at *P*=.02.

[as]The difference between Black and multiracial/other race women is statistically significant at *P*=.03.

[at]The difference between Latinx and multiracial/other race women is statistically significant at *P*=.01.

[au]The difference between White and AIAN women is statistically significant at *P*=.003.

[av]Includes temporary/transitional housing, single room occupancy hotel, shelter, drug treatment housing, abandoned building, and street.

[aw]The difference between Latinx and AIAN women is statistically significant at *P*=.01.
