# Supplementary material for: Racial and Ethnic Differences in COVID-19 Outcomes, Stressors, Fear, and Prevention Behaviors Among US Women: Web-Based Cross-sectional Study
Source: J Med Internet Res. 2021 Jul 12;23(7):e26296. doi: 10.2196/26296 (PMC8276781; doi:10.2196/26296)
Supplement: Multimedia Appendix 2 [file jmir_v23i7e26296_app2.pdf]

**Multimedia Appendix 2.** COVID-19 outcomes by racial/ethnic group among adult women in the United States (N=473).[a]

| Outcome | | Overall (N=473), n (%) | White (n=241), n (%) | API[b] (n=64), n (%) | Black (n=60), n (%) | Latinx (n=48), n (%) | AIAN[c] (n=27), n (%) | Multiracial or other (n=33), n (%) | *P* value |
|---|---|---|---|---|---|---|---|---|---|
| Have had COVID-19 symptoms | | 73 (15.5) | 45 (18.8) | 4 (6.4) | 6 (10.0) | 8 (17.0) | 3 (11.1) | 7 (21.2) | .12 |
| Been tested for COVID-19 | | 27 (5.8) | 16 (6.7) | 3 (4.7) | 3 (5.0) | 3 (6.3) | 2 (7.4) | 0 (0) | .77 |
| Know where to go to get tested for COVID-19 | | 227 (51.4) | 133 (59.9)[d,e,f] | 21 (34.4)[d,g] | 33 (57.9)[g,h] | 18 (40.0)[e] | 8 (32.0)[f,h] | 14 (43.8) | <.001 |
| Had a positive COVID-19 test result | | 8 (1.7) | 3 (1.3) | 0 (0) | 3 (5.1) | 0 (0) | 1 (3.7) | 1 (3.0) | .13 |
| Hospitalized for COVID-19 treatment[i] | | 4 (50.0) | 1 (33.3) | 0 (0) | 1 (33.3) | 0 (0) | 1 (100.0) | 1 (100.0) | >.99 |
| Had someone close to you test positive for COVID-19 | | 82 (17.5) | 51 (21.3)[j] | 5 (7.9)[j,k] | 14 (23.3)[k] | 7 (14.9) | 2 (7.7) | 3 (9.1) | .05 |
| **Relationship of people close to you who tested positive for COVID-19[l]** | | | | | | | | | |
| | Family | 35 (42.7) | 19 (37.3) | 1 (20.0) | 11 (78.6) | 2 (28.6) | 1 (50.0) | 1 (33.3) | .05 |
| | Friend | 55 (67.1) | 36 (70.6) | 2 (40.0) | 6 (42.9) | 6 (85.7) | 2 (100.0) | 3 (100.0) | .11 |
| | Partner | 2 (2.4) | 1 (2.0) | 0 (0) | 0 (0) | 0 (0) | 1 (50.0) | 0 (0) | .17 |
| | Coworker | 15 (18.3) | 6 (11.8) | 1 (20.0) | 4 (28.6) | 3 (42.9) | 1 (50.0) | 0 (0) | .12 |
| | Other[m] | 1 (1.2) | 0 (0) | 0 (0) | 0 (0) | 0 (0) | 0 (0) | 1 (33.3) | N/A[n] |
| Had someone close to you hospitalized for COVID-19[l] | | 35 (42.7) | 15 (29.4)[o] | 2 (40.0) | 11 (78.6)[o] | 3 (42.9) | 2 (100.0) | 2 (66.7) | .004 |
| **Relationship of people close to you hospitalized for COVID-19[p]** | | | | | | | | | |

| Outcome | | Overall (N=473), n (%) | White (n=241), n (%) | API[b] (n=64), n (%) | Black (n=60), n (%) | Latinx (n=48), n (%) | AIAN[c] (n=27), n (%) | Multiracial or other (n=33), n (%) | *P* value |
|---|---|---|---|---|---|---|---|---|---|
| | Family | 16 (45.7) | 4 (26.7)[q] | 0 (0) | 9 (81.8)[q] | 2 (66.7) | 1 (50.0) | 0 (0) | .01 |
| | Friend | 14 (40.0) | 6 (40.0) | 1 (50.0) | 2 (18.2) | 2 (66.7) | 1 (50.0) | 2 (100.0) | .23 |
| | Coworker | 7 (20.0) | 3 (20.0) | 1 (50.0) | 2 (18.2) | 1 (33.3) | 0 (0) | 0 (0) | .87 |
| | Other[r] | 3 (8.6) | 3 (20.0) | 0 (0) | 0 (0) | 0 (0) | 0 (0) | 0 (0) | .61 |
| Had someone close to you die from COVID-19[l] | | 18 (22.0) | 7 (13.7) | 1 (20.0) | 5 (35.7) | 2 (28.6) | 1 (50.0) | 2 (66.7) | .08 |
| **Relationship of people you know who have died from COVID-19**[s] | | | | | | | | | |
| | Family | 6 (33.3) | 1 (14.3) | 0 (0) | 3 (60.0) | 2 (100.0) | 0 (0) | 0 (0) | .15 |
| | Friend | 8 (44.4) | 2 (28.6) | 0 (0) | 2 (40.0) | 1 (50.0) | 1 (100.0) | 2 (100.0) | .54 |
| | Coworker | 4 (22.2) | 1 (14.3) | 1 (100.0) | 2 (40.0) | 0 (0) | 0 (0) | 0 (0) | .51 |
| | Other[t] | 3 (16.7) | 3 (42.9) | 0 (0) | 0 (0) | 0 (0) | 0 (0) | 0 (0) | N/A |
| Concerned that you may have been exposed to COVID-19 by these people you know[l] | | 18 (22.0) | 9 (17.7) | 2 (40.0) | 3 (21.4) | 2 (28.6) | 1 (50.0) | 1 (33.3) | .46 |

[a]Certain percentages may reflect denominators smaller than the n value given in the column heading. These discrepancies are due to missing data.

[b]API: Asian, Native Hawaiian, or other Pacific Islander.

[c]AIAN: American Indian or Alaskan Native.

[d]The difference between White and API women is statistically significant at $P<.001$.

[e]The difference between White and Latinx women is statistically significant at *P*=.01.

[f]The difference between White and AIAN women is statistically significant at *P*=.01.

[g]The difference between API and Black women is statistically significant at *P*=.01.

[h]The difference between Black and AIAN women is statistically significant at *P*=.03.

[i]Percentages correspond with the 8 women who tested positive for COVID-19.

[j]The difference between White and API women is statistically significant at *P*=.02.

[k]The difference between API and Black women is statistically significant at *P*=.02.

[l]Percentages correspond with the 82 women who knew someone close who tested positive for COVID-19.

[m]Includes two children in their home daycare.

[n]N/A: not applicable.

[o]The difference between White and Black women is statistically significant at *P*<.001.

[p]Percentages correspond with the 35 women who knew someone close to them who had been hospitalized for COVID-19.

[q]The difference between White and Black women is statistically significant at *P*=.01.

[r]Includes friend's dad, neighbor, and child of acquaintance.

[s]Percentages correspond with the 18 women who knew someone close to them who died from COVID-19.

[t]Includes childhood acquaintances, friend's dad, neighbor, and relative of friend.
