# Supplementary material for: Racial and Ethnic Differences in COVID-19 Outcomes, Stressors, Fear, and Prevention Behaviors Among US Women: Web-Based Cross-sectional Study
Source: J Med Internet Res. 2021 Jul 12;23(7):e26296. doi: 10.2196/26296 (PMC8276781; doi:10.2196/26296)
Supplement: Multimedia Appendix 3 [file jmir_v23i7e26296_app3.pdf]

Multimedia Appendix 3. COVID-19 stressors and fear by racial/ethnic group among adult women in the United States (N=473).[a]

Characteristic	Overall (N=473)	White (n=241)	API[b] (n=64)	Black (n=60)	Latinx (n=48)	AIAN[c] (n=27)	Multiracial or other (n=33)	*P* value
**COVID-19–related stressors, n (%)**								
Not enough food and no money to buy more	82 (17.3)	18 (7.5)[d,e,f,g,h]	23 (35.9)[d,i]	14 (23.3)[e]	9 (18.8)[f,i]	9 (33.3)[g]	9 (27.3)[h]	<.001
Not enough money to pay rent	91 (19.2)	24 (10.0)[d,e,f,g]	23 (35.9)[d]	17 (28.3)[e]	10 (20.8)[f]	10 (37.0)[g]	7 (21.2)	<.001
Taking care of a sick family member or friend without COVID-19	47 (9.9)	29 (12.0)	2 (3.1)	9 (15.0)	2 (4.2)	2 (7.4)	3 (9.1)	.13
Taking care of a sick family member or friend with or possibly with COVID-19	15 (3.2)	7 (2.9)	2 (3.1)	2 (3.3)	1 (2.1)	2 (7.4)	1 (3.0)	.79
Homeschooling children	99 (20.9)	61 (25.3)[j,k]	8 (12.5)[j]	14 (23.3)[l]	11 (22.9)[m]	0 (0)[k,l,m]	5 (15.2)	.02
Not being able to go to the doctor or have a phone/video appointment	133 (28.1)	85 (35.3)[n,o]	10 (15.6)[n]	15 (25.0)	10 (20.8)	4 (14.8)[o]	9 (27.3)	.01
Not being able to get important medication	22 (4.7)	11 (4.6)	2 (3.1)	2 (3.3)	3 (6.3)	0 (0)	4 (12.1)	.35
Loss of income	173 (36.6)	96 (39.8)	19 (29.7)	21 (35.0)	15 (31.3)	7 (25.9)	15 (45.5)	.36
Going to work as an essential employee	73 (15.4)	40 (16.6)	6 (9.4)	14 (23.3)	8 (16.7)	2 (7.4)	3 (9.1)	.20
Other[p]	26 (5.5)	17 (7.1)[q]	3 (4.7)	0 (0)[q,r]	1 (2.1)	1 (3.7)	4 (12.1)[r]	.09

Characteristic		Overall (N=473)	White (n=241)	API[b] (n=64)	Black (n=60)	Latinx (n=48)	AIAN[c] (n=27)	Multiracial or other (n=33)	*P* value
	None	99 (20.9)	50 (20.8)	12 (18.8)	10 (16.7)	12 (25.0)	8 (29.6)	7 (21.2)	.77
**Fear of coronavirus[s], median (IQR)**		22 (16-26)	22 (16-26)	21 (17-25.5)	21 (15-24)	22 (15-28)	24 (16-34)	18 (15-24)	.21
	Afraid of losing life because of COVID-19	3 (2-4)	3 (2-4)	3 (2-4)	3 (2-4)	3 (2-4)	3 (2-5)	2 (2-4)	.38
	Cannot sleep due to worry about getting COVID-19	3 (2-4)	2 (1-4)[t]	3 (2-4)[u]	2 (1-3)[u,v]	3 (1-4)	3 (2-5)[t,v,w]	2 (2-3)[w]	<.001
	Heart races when thinking about it	3 (2-4)	2 (1-4)	3 (2-4)	2 (1.5-3)[x]	3 (1-4)	3 (2-5)[x]	3 (2-3)	.02
	Afraid of COVID-19	4 (3-4)	4 (3-4)[y,z]	3 (2-4)[y]	4 (3-4)	4 (3-5)	4 (2-5)	3 (2-4)[z]	.002
	Become nervous or anxious when watching news stories on social media	4 (3-4)	4 (3-4.5)[aa]	3 (2.5-4)[aa]	4 (3-4)	4 (2-4)	4 (3-5)	4 (3-4)	.004
	Hands become clammy thinking about it	2 (1-3)	2 (1-3)[ab,ac]	3 (2-4)	2 (1-3)[ad]	3 (2-4)[ab,ae]	3 (2-5)[ac,ad,af]	2 (1-2)[ae,af]	<.001
	Uncomfortable thinking about COVID-19	4 (2-4)	4 (3-4)	3 (2-4)	3 (2-4)	3 (2-4)	4 (2-5)	3 (3-4)	.05

[a]Certain percentages may reflect denominators smaller than the n value given in the column heading. These discrepancies are due to missing data.

[b]API: Asian, Native Hawaiian, or other Pacific Islander.

[c]AIAN: American Indian or Alaskan Native.

[d]The difference between White and API women is statistically significant at $P<.001$.

[e]The difference between White and Black women is statistically significant at $P<.001$.

[f]The difference between White and Latinx women is statistically significant at $P=.03$.

[g]The difference between White and AIAN women is statistically significant at $P<.001$.

[h]The difference between White and multiracial/other race women is statistically significant at $P=.002$.

[i]The difference between API and Latinx women is statistically significant at $P=.046$.

[j]The difference between White and API women is statistically significant at $P=.03$.

[k]The difference between White and AIAN women is statistically significant at $P=.003$.

[l]The difference between Black and AIAN women is statistically significant at $P=.004$.

[m]The difference between Latinx and AIAN women is statistically significant at $P=.01$.

[n]The difference between White and API women is statistically significant at $P=.003$.

[o]The difference between White and AIAN women is statistically significant at $P=.03$.

[p]Do not have text responses for this *other* variable.

[q]The difference between White and Black women is statistically significant at $P=.03$.

[r]The difference between Black and multiracial/other race women is statistically significant at $P=.01$.

[s]7-item, four-point Likert scale that includes questions such as whether a participant is afraid of COVID-19, uncomfortable thinking about COVID-19, hands become clammy thinking about it, afraid of losing life because of COVID-19, become nervous or anxious when watching news stories on social media, cannot sleep due to worry about getting COVID-19, and heart races when thinking about it.

[t]The difference between White and AIAN women is statistically significant at $P=.01$.

[u]The difference between API and Black women is statistically significant at *P*=.02.

[v]The difference between Black and AIAN women is statistically significant at *P*=.002.

[w]The difference between AIAN and multiracial/other race women is statistically significant at *P*=.01.

[x]The difference between Black and AIAN women is statistically significant at *P*=.04.

[y]The difference between White and API women is statistically significant at *P*=.01.

[z]The difference between White and multiracial/other race women is statistically significant at *P*=.02.

[aa]The difference between White and API women is statistically significant at *P*=.001.

[ab]The difference between White and Latinx women is statistically significant at *P*=.02.

[ac]The difference between White and AIAN women is statistically significant at *P*=.002.

[ad]The difference between Black and AIAN women is statistically significant at *P*=.03.

[ae]The difference between Latinx and multiracial/other race women is statistically significant at *P*=.02.

[af]The difference between AIAN and multiracial/other race women is statistically significant at *P*=.002.
