# Supplementary material for: Racial and Ethnic Differences in COVID-19 Outcomes, Stressors, Fear, and Prevention Behaviors Among US Women: Web-Based Cross-sectional Study
Source: J Med Internet Res. 2021 Jul 12;23(7):e26296. doi: 10.2196/26296 (PMC8276781; doi:10.2196/26296)
Supplement: Multimedia Appendix 4 [file jmir_v23i7e26296_app4.pdf]

Multimedia Appendix 4. COVID-19 public health prevention behaviors by racial/ethnic group among adult women in the United States (N=473).[a]

Prevention behavior	Overall (N=473), n (%)	White (n=241), n (%)	API[b] (n=64), n (%)	Black (n=60), n (%)	Latinx (n=48), n (%)	AIAN[c] (n=27), n (%)	Multiracial or other (n=33), n (%)	*P* value
Washing hands with soap	452 (95.6)	235 (97.5)[d]	62 (96.9)[e]	56 (93.3)	46 (95.8)	22 (81.5)[d,e]	31 (93.9)	.01
Avoiding touching your face	374 (79.1)	181 (75.1)	56 (87.5)	48 (80.0)	43 (89.6)	21 (77.8)	25 (75.8)	.13
Using disinfectants	412 (87.1)	212 (88.0)	57 (89.1)	53 (88.3)	38 (79.2)	24 (88.9)	28 (84.9)	.64
Staying home except for essential activities	419 (88.6)	220 (91.3)	58 (90.6)	52 (86.7)	36 (75.0)	24 (88.9)	29 (87.9)	.05
Covering your mouth when you cough	389 (82.2)	203 (84.2)	55 (85.9)	48 (80.0)	34 (70.8)	21 (77.8)	28 (84.9)	.28
Physical distancing or staying 6 feet away from people outside your household	409 (86.5)	212 (88.0)	59 (92.2)	52 (86.7)	36 (75.0)	21 (77.8)	29 (87.9)	.09
Physical distancing or staying 6 feet away from all others if you are sick	283 (59.8)	138 (57.3)	47 (73.4)	33 (55.0)	31 (64.6)	15 (55.6)	19 (57.6)	.23
Isolating yourself at home and away from others if you are sick	284 (60.0)	143 (59.3)[f]	49 (76.6)[f,g,h]	31 (51.7)[g]	29 (60.4)	17 (63.0)	15 (45.5)[h]	.03
Using a face mask in public	377 (79.7)	196 (81.3)	51 (79.7)	48 (80.0)	34 (70.8)	20 (74.1)	28 (84.9)	.58
Using gloves in public	190 (40.2)	72 (29.9)[i,j,k]	46 (71.9)[i,l,m,n]	26 (43.3)[j,l,o]	18 (37.5)[m,p]	19 (70.4)[k,o,p,q]	9 (27.3)[n,q]	<.001

Prevention behavior	Overall (N=473), n (%)	White (n=241), n (%)	API[b] (n=64), n (%)	Black (n=60), n (%)	Latinx (n=48), n (%)	AIAN[c] (n=27), n (%)	Multiracial or other (n=33), n (%)	*P* value
Avoiding crowds	387 (81.8)	208 (86.3)	53 (82.8)	47 (78.3)	34 (70.8)	19 (70.4)	26 (78.8)	.07
Not leaving your home for any activities	206 (43.6)	80 (33.2)[i,r,s]	45 (70.3)[i,t,u]	27 (45.0)[t]	19 (39.6)[u,v]	18 (66.7)[r,v]	17 (51.5)[s]	<.001
Other[w]	1 (0.2)	0 (0)	0 (0)	0 (0)	0 (0)	0 (0)	1 (3.0)	N/A[x]

[a]Certain percentages may reflect denominators smaller than the n value given in the column heading. These discrepancies are due to missing data.

[b]API: Asian, Native Hawaiian, or other Pacific Islander.

[c]AIAN: American Indian or Alaskan Native.

[d]The difference between White and AIAN women is statistically significant at *P*=.002.

[e]The difference between API and AIAN women is statistically significant at *P*=.02.

[f]The difference between White and API women is statistically significant at *P*=.01.

[g]The difference between API and Black women is statistically significant at *P*=.004.

[h]The difference between API and multiracial/other race women is statistically significant at *P*=.002.

[i]The difference between White and API women is statistically significant at *P*<.001.

[j]The difference between White and Black women is statistically significant at *P*=.047.

[k]The difference between White and AIAN women is statistically significant at *P*<.001.

[l]The difference between API and Black women is statistically significant at *P*=.002.

[m]The difference between API and Latinx women is statistically significant at *P*<.001.

[n]The difference between API and multiracial/other race women is statistically significant at *P*<.001.

[o]The difference between Black and AIAN women is statistically significant at *P*=.02.

[p]The difference between Latinx and AIAN women is statistically significant at *P*=.01.

[q]The difference between AIAN and multiracial/other race women is statistically significant at *P*<.001.

[r]The difference between White and AIAN women is statistically significant at *P*=.001.

[s]The difference between White and multiracial/other race women is statistically significant at *P*=.04.

[t]The difference between API and Black women is statistically significant at *P*=.01.

[u]The difference between API and Latinx women is statistically significant at *P*=.001.

[v]The difference between Latinx and AIAN women is statistically significant at *P*=.02.

[w]Do not have text responses for this *other* variable.

[x]N/A: not applicable.
