# Supplementary material for: Racial and Ethnic Differences in COVID-19 Outcomes, Stressors, Fear, and Prevention Behaviors Among US Women: Web-Based Cross-sectional Study
Source: J Med Internet Res. 2021 Jul 12;23(7):e26296. doi: 10.2196/26296 (PMC8276781; doi:10.2196/26296)
Supplement: Multimedia Appendix 5 [file jmir_v23i7e26296_app5.pdf]

Multimedia Appendix 5. Unadjusted and adjusted binary logistic regression models of COVID-19 related outcomes, stressors, and prevention behaviors and racial/ethnic group among adult women in the United States (N=473).

	Asian/ Pacific Islander[a]		Black[a]		Latinx[a]		American Indian/ Alaskan Native[a]		Multiracial & Other[a]	
Characteristic	OR[b] (95% CI)	AOR[c] (95% CI)	OR[b] (95% CI)	AOR[c] (95% CI)	OR[b] (95% CI)	AOR[c] (95% CI)	OR[b] (95% CI)	AOR[c] (95% CI)	OR[b] (95% CI)	AOR[c] (95% CI)
**COVID-19 Related Outcomes**										
Know where to go to get tested for coronavirus	0.35 (0.19-0.64)	0.36 (0.19-0.70)	0.92 (0.51-1.66)	0.88 (0.46-1.68)	0.45 (0.23-0.86)	0.53 (0.26-1.06)	0.32 (0.13-0.76)	0.26 (0.09-0.72)	0.52 (0.25-1.10)	0.56 (0.24-1.27)
Had someone close to you hospitalized for coronavirus	0.32 (0.12-0.84)	0.51 (0.18-1.42)	1.13 (0.58-2.21)	1.45 (0.68-3.10)	0.65 (0.27-1.53)	0.96 (0.39-2.37)	0.31 (0.07-1.35)	0.45 (0.10-2.11)	0.37 (0.11-1.26)	0.56 (0.16-2.03)
Family member hospitalized for coronavirus	0.19 (0.02-1.41)	0.24 (0.03-1.97)	2.62 (1.17-5.86)	3.24 (1.30-8.07)	0.51 (0.11-2.26)	0.60 (0.13-2.79)	0.45 (0.06-3.50)	0.53 (0.06-4.58)	0.37 (0.05-2.82)	0.47 (0.06-3.91)
**COVID-19 Related Stressors**										
Not enough food and no money to buy more	6.95 (3.45-14.01)	4.46 (1.90-10.49)	3.77 (1.75-8.12)	3.24 (1.28-8.20)	2.86 (1.20-6.82)	2.44 (0.83-7.15)	6.19 (2.44-15.75)	3.58 (1.19-10.81)	4.65 (1.88-11.48)	4.26 (1.44-12.59)
Not enough money to pay rent	5.07 (2.62-9.84)	3.03 (1.39-6.63)	3.58 (1.77-7.21)	2.67 (1.15-6.20)	2.38 (1.05-5.37)	1.34 (0.49-3.64)	5.32 (2.19-12.92)	2.96 (1.06-8.27)	2.43 (0.96-6.20)	1.47 (0.50-4.36)
Homeschooling children	0.42 (0.19-0.93)	0.71 (0.29-1.76)	0.90 (0.46-1.75)	1.30 (0.61-2.79)	0.88 (0.42-1.83)	1.09 (0.48-2.47)	N/A[d]	N/A[d]	0.53 (0.20-1.43)	1.11 (0.38-3.30)
Not being able to go to the doctor or have a phone/video appointment	0.34 (0.17-0.70)	0.40 (0.18-0.87)	0.61 (0.32-1.16)	0.74 (0.37-1.48)	0.48 (0.23-1.02)	0.61 (0.28-1.32)	0.32 (0.11-0.95)	0.27 (0.08-0.98)	0.69 (0.31-1.55)	0.78 (0.33-1.87)
Cannot sleep due to worry about getting coronavirus	1.28 (1.03-1.60)	1.31 (1.01-1.70)	0.83 (0.65-1.06)	0.80 (0.61-1.04)	1.15 (0.90-1.48)	1.02 (0.78-1.35)	1.79 (1.28-2.50)	1.79 (1.21-2.65)	0.88 (0.65-1.19)	0.87 (0.62-1.23)
Heart races when thinking about it	1.22 (0.98-1.51)	1.20 (0.93-1.54)	0.86 (0.69-1.09)	0.82 (0.64-1.06)	1.03 (0.80-1.31)	0.91 (0.70-1.19)	1.53 (1.11-2.11)	1.45 (1.01-2.09)	0.94 (0.70-1.25)	0.92 (0.67-1.27)
Afraid of coronavirus	0.65 (0.51-0.82)	0.78 (0.60-1.01)	0.80 (0.63-1.02)	0.88 (0.67-1.15)	0.88 (0.67-1.15)	0.91 (0.68-1.22)	0.80 (0.57-1.12)	0.94 (0.64-1.38)	0.65 (0.48-0.87)	0.75 (0.53-1.06)
Become nervous or anxious when watching news	0.70 (0.56-0.88)	0.74 (0.57-0.96)	0.88 (0.70-1.12)	0.92 (0.71-1.21)	0.84 (0.65-1.08)	0.84 (0.64-1.11)	0.95 (0.68-1.33)	0.94 (0.63-1.40)	0.85 (0.63-1.15)	0.82 (0.58-1.16)

stories on social media										
Hands become clammy thinking about it	1.33 (1.07-1.66)	1.29 (0.99-1.66)	1.08 (0.85-1.36)	1.04 (0.80-1.36)	1.47 (1.15-1.88)	1.28 (0.98-1.67)	1.91 (1.39-2.63)	1.94 (1.32-2.84)	0.81 (0.58-1.13)	0.81 (0.56-1.16)
**COVID-19 Prevention Behaviors**										
Washing hands with soap	0.79 (0.16-4.02)	1.05 (0.19-5.78)	0.36 (0.10-1.31)	0.50 (0.13-1.95)	0.59 (0.12-3.00)	N/A[e]	0.11 (0.03-0.40)	0.17 (0.04-0.71)	0.40 (0.08-2.05)	0.57 (0.10-3.20)
Isolating yourself at home and away from others if you are sick	2.24 (1.19-4.22)	2.21 (1.08-4.53)	0.73 (0.42-1.29)	0.64 (0.34-1.20)	1.05 (0.56-1.97)	1.04 (0.52-2.08)	1.17 (0.51-2.65)	0.96 (0.38-2.37)	0.57 (0.28-1.19)	0.52 (0.23-1.18)
Using gloves in public	6.00 (3.26-11.05)	4.99 (2.52-9.88)	1.80 (1.01-3.21)	1.53 (0.80-2.93)	1.41 (0.74-2.69)	1.28 (0.63-2.59)	5.57 (2.33-13.32)	4.05 (1.58-10.37)	0.88 (0.39-1.99)	0.74 (0.30-1.81)
Not leaving your home for any activities	4.77 (2.62-8.68)	5.13 (2.58-10.18)	1.65 (0.93-2.93)	1.57 (0.81-3.03)	1.32 (0.70-2.50)	1.30 (0.63-2.65)	4.03 (1.73-9.36)	3.85 (1.52-9.75)	2.14 (1.03-4.45)	1.95 (0.85-4.44)

[a]Reference for the dependent variable of race is ‘White’ for each of the racial/ethnic group comparisons.

[b]All unadjusted models included race and one independent variable.

[c]All adjusted models included race and one independent variable, while adjusting for age, education, income, and type of residential community.

[d]Zero ‘Checked’ responses for ‘Homeschooling children’ for American Indian or Alaskan Native respondents.

[e]Zero ‘Unchecked’ responses for ‘Washing hands with soap’ for Latinx respondents.
